# A Label-Free Impedance Immunosensor Using Screen-Printed Interdigitated Electrodes and Magnetic Nanobeads for the Detection of *E. coli* O157:H7

**DOI:** 10.3390/bios5040791

**Published:** 2015-12-15

**Authors:** Ronghui Wang, Jacob Lum, Zach Callaway, Jianhan Lin, Walter Bottje, Yanbin Li

**Affiliations:** 1 Department of Biological and Agricultural Engineering, University of Arkansas, Fayetteville, AR 72701, USA; E-Mails: rwang@uark.edu (R.W.); zcallawa@uark.edu (Z.C.); 2 Cell and Molecular Biology Program, University of Arkansas, Fayetteville, AR 72701, USA; E-Mail: jlum@pacificvetgroup.com; 3 College of Information and Electrical Engineering, China Agricultural University, Beijing 100083, China; E-Mail: jianhan@cau.edu.cn; 4 Center of Excellence for Poultry Science, University of Arkansas, Fayetteville, AR 72701, USA; E-Mail: wbottje@uark.edu; 5 College of Biosystems Engineering and Food Science, Zhejiang University, Hangzhou 310068, China

**Keywords:** immunosensor, magnetic nanobeads, impedance, screen-printed interdigitated electrode, *E. coli* O157:H7, rapid detection

## Abstract

*Escherichia coli* O157:H7 is one of the leading bacterial pathogens causing foodborne illness. In this study, an impedance immunosensor based on the use of magnetic nanobeads and screen-printed interdigitated electrodes was developed for the rapid detection of *E. coli* O157:H7. Magnetic nanobeads coated with anti-*E. coli* antibody were mixed with an *E. coli* sample and used to isolate and concentrate the bacterial cells. The sample was suspended in redox probe solution and placed onto a screen-printed interdigitated electrode. A magnetic field was applied to concentrate the cells on the surface of the electrode and the impedance was measured. The impedance immunosensor could detect *E. coli* O157:H7 at a concentration of 10^4.45^ cfu·mL^−1^ (~1400 bacterial cells in the applied volume of 25 μL) in less than 1 h without pre-enrichment. A linear relationship between bacteria concentration and impedance value was obtained between 10^4^ cfu·mL^−1^ and 10^7^ cfu·mL^−1^. Though impedance measurement was carried out in the presence of a redox probe, analysis of the equivalent circuit model showed that the impedance change was primarily due to two elements: Double layer capacitance and resistance due to electrode surface roughness. The magnetic field and impedance were simulated using COMSOL Multiphysics software.

## 1. Introduction

*Escherichia coli* O157:H7 is one of the most dangerous foodborne pathogens, infecting an estimated 63,000 people in the US each year, including 20 deaths, and having an infective dose as low as 10 cells [1,2]. Infection of *E. coli* O157:H7 may cause a life-threatening complication known as hemolytic uremic syndrome in 10%–15% of patients with hemorrhagic colitis. *E. coli* O157:H7 infections have primarily been associated with ground beef and leafy green produce but increased integration of the food supply chain has resulted in *E. coli* O157:H7 contamination of unusual food products, such as cookie dough and hazelnuts [3]. Contaminated food products not only threaten human health but also cost food producers millions of dollars in economic loss [4]. As such, a method to rapidly detect *E. coli* O157:H7 in food products is needed.

Bacterial culture and plating and polymerase chain reaction are the traditional methods for *E. coli* O157:H7 detection, but these methods are time-consuming and require trained personnel and specialized laboratories and equipment. Results may take days, during which food products may have been shipped to consumers or to other producers. Biosensors have attracted attention in the field of foodborne pathogen detection due to their speed, simplicity, and low cost. Several types of biosensors have been developed for the detection of *E. coli* O157:H7 including quartz crystal microbalance [5,6,7,8], surface plasmon resonance [9,10,11,12] and electrochemistry [13,14,15,16,17,18,19,20,21]. Many of the developed biosensors relied on immobilization of antibodies on the sensing surface to concentrate and hold the bacterial cells close enough to the sensing surface for measurement. This method has the problem of low capture efficiency, often being as low as 35% even after extensive optimization [22]. A method not reliant on electrode immobilization should be used to overcome the problem of low capture efficiency.

Magnetic nanoparticles have been used extensively in biosensors for bacterial detection though usually for immunomagnetic separation of the bacteria from a sample [13,14,15] or as labels to increase the sensitivity of the biosensor [7,8]. Magnetic nanoparticles may also be used to concentrate the bacterial cells onto the sensing surface, as done by Varshney and Li [13], where a magnetic field was applied under the electrode to pull the bacteria close to an interdigitated microelectrode array for sensitive detection. The interdigitated microelectrode arrays used by Varshney and Li [13], while being highly sensitive, were time-consuming and expensive to produce, making them impractical for commercial use. Screen printed interdigitated electrodes are capable of being produced at a much lower cost and in high volume, making them practical for use in commercialized rapid tests. Recently, a screen printed interdigitated electrode was successfully used for the development of an impedance biosensor for detection of avian influenza (AI) virus [23], but this reported biosensor required signal amplification with labels. The impedance immunosensor developed in this research was a label-free detection approach. In addition, the size of AI virus is 80–120 nm in diameter, whereas an *E. coli* O157:H7 cell is about 1–1.5 μm long and 0.5 μm (or 500 nm) in diameter, and there are many differences in biological components; e.g. physical structure and binding sites, between AI virus and bacterial cells. Therefore, we have attempted to explore a new application of the screen printed interdigitated electrode based impedance immunosensor for bacteria detection.

In this study, an impedance immunosensor for the detection of *E. coli* O157:H7 was developed using antibody-coated magnetic nanobeads and screen printed interdigitated electrodes. In the research the antibody-coated magnetic nanobeads served three roles: (1) to specifically separate *E. coli* O157:H7 cells from media and place them in redox probe for measurement; (2) to concentrate the separated *E. coli* O157:H7 into a smaller volume; and (3) to concentrate the *E. coli* O157:H7 cells onto the surface of the screen printed electrode. An equivalent circuit model was developed to understand the phenomenon involved in the impedance measurement.

## 2. Experimental Section

### 2.1. Bacterial Culture

*Escherichia coli* O157:H7 was purchased from American Type Culture Collection (ATCC 43888) and stored in brain heart infusion broth (BHI, Remel Inc., Lenexa, KS, USA) at −80 °C. The culture was grown in brain-heart infusion (BHI) broth at 37 °C for 18 h. For enumeration the culture was serially diluted in phosphate buffered saline (PBS; 0.01 M; pH 7.4; Sigma-Aldrich, St. Louis, MO, USA) and plated on sorbitol MacConkey agar (SMAC, Remel Inc., part of Thermo Fisher Scientific, Lenexa, KS, USA) incubated at 37 °C for 22–24 h. Due to biosafety concerns, the bacteria was killed by boiling for 10 min before use in biosensor tests.

### 2.2. Biological and Chemical Reagents

Phosphate buffered saline (PBS; 0.01 M; pH 7.4) was purchased from Sigma-Aldrich. Biotin-labeled anti-*E. coli* antibody (Catalog # B65007R) was purchased from Meridian Life Science (Memphis, TN, USA). It is a rabbit antibody to *E. coli*, O and K antigenic serotypes. It was diluted to 0.4–0.5 mg·mL^−1^ with PBS for use in tests. All solutions were prepared with deionized water from Millipore (Milli-Q, 18.2 MΩ·cm, Bedford, MA, USA).

### 2.3. Screen-Printed Interdigitated Electrodes and Magnetic Nanobeads

Gold screen-printed interdigitated electrodes were provided by Aibit, LLC (Jiangyin, China). The interdigitated electrode consisted of three pairs of 200 µm wide electrodes spaced 200 µm apart arranged in a circular array printed on a ceramic substrate. The outer diameter of the array was 5.4 mm. A photograph and drawing of the interdigitated screen printed electrode is shown in the supplemental materials (Figure S1). Streptavidin coated magnetic nanobeads (Fe_3_O_4_; ~150 nm diameter) were purchased from R & D Systems (Minneapolis, MN, USA) and used at the stock concentration (1 mg·mL^−1^).

### 2.4. Impedance Measurement 

Impedance measurements were performed using an IM-6 impedance analyzer with IM-6/Thales software (BAS, West Lafayette, IN, USA). Test-sense and counter-reference probes were connected to the electrode. An AC potential of 50 mV was used for all impedance measurements. Impedance magnitude and phase angle were measured at 38 points in the frequency range of 10 Hz to 100 kHz. All impedance measurements were done in the presence of a redox probe consisting of 5 mM [Fe(CN)_6_]^3−/4−^ (1:1 ratio) mixture in PBS.

### 2.5. Immunomagnetic Separation of E. coli O157:H7

Antibody-coated nanobeads were prepared by mixing 20 μL of streptavidin coated magnetic nanobeads with 20 μL of biotin labeled anti- *E. coli* O157:H7 antibody in 200 μL of PBS for 45 min in a rotating mixer at 5 rpm. A magnetic field (~0.7 T) was applied using a magnetic separator consisting of six permanent magnets (Aibit LLC) for 4 min and the bead/antibody complexes were washed twice with 200 μL of PBS. The nanobeads were split into two tubes. A 200 μL sample of *E. coli* O157:H7 was added to one tube and 200 μL of PBS was added to another tube to serve as a negative control sample. The samples were mixed for 45 min in a rotating mixer at 5 rpm. The samples were then magnetically separated for 4 min and washed twice with redox probe and each suspended in 100 μL of redox probe for impedance measurement. All mixing was done at room temperature.

### 2.6. Detection of E. coli O157:H7

**Figure 1:**
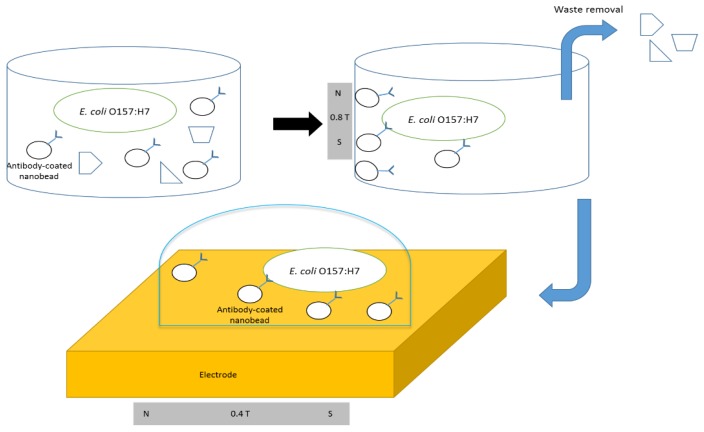
Immunomagnetic separation of *E. coli* O157:H7 from media using the antibody-coated nanobeads and the concentration of bacteria on the electrode surface using a magnetic field.

Screen-printed interdigitated electrodes were cleaned using 1 M NaOH for 3 min. The electrode was then washed with deionized water and dried with nitrogen. Impedance detection of *E. coli* O157:H7 was done by placing a 25 μL drop of a prepared sample from immunomagnetic separation onto the electrode surface. A magnetic field with a surface intensity of ~0.4 T was applied using a permanent magnet (neodymium magnet, CMS Magnetics, Garland, TX, USA) and used to draw the bacteria/nanobead complexes to the electrode surface for 10 min before impedance measurement. The impedance was measured while the magnetic field was still being applied. The impedance of the control sample prepared in parallel with the bacterial sample was measured first to gather a baseline for detection. The impedance of each bacteria sample was compared to the control sample prepared in parallel to it. Figure 1 shows the diagrams of the immunomagnetic separation and detection protocols.

### 2.7. The Preparation, Inoculation and Detection of Ground Beef Samples

Ground beef was purchased from a local grocery store, and transported to the lab within 15 min. Twenty-five grams of ground beef was weighted and transferred into a filtering stomacher bag. Then, 225 mL of sterile PBS solution was added to the stomacher bag, and mixed in a stomacher (Stomacher 400, Seward, UK) at 200 rpm for two min. After that, 10 mL of stomaching solution were collected and inoculated with *E. coli* O157:H7 at concentrations of 10^5^ and 10^6^ cfu·mL^−1^ for use in the tests. The same protocol as described earlier for detection of *E. coli* O157:H7 was followed in the test, except the ground beef sample was used instead of pure bacterial culture of *E coli* O157:H7.

### 2.8. Equivalent Circuit Modeling and Statistical Analysis

An equivalent circuit was built and evaluated using IM-6/Thales software. Statistical analysis of data and preparation of graphs were done using Microsoft Excel (Microsoft, Redmond, WA, USA).

*Escherichia coli* O157:H7 at concentrations of 10^4^ to 10^7^ cfu·mL^−1^ were measured. Means and standard deviations were calculated based on triplicate tests. Lower detection limits were determined as a signal/noise ratio of 3, where the noise was defined as the standard deviation of the negative PBS control. Statistically significant differences were determined using t-tests (α = 0.05).

### 2.9. Electron Microscopy

Electron scanning electron microscopy was done using a Philips XL30 ESEM (Environmental Scanning Electron Microscope, FEI, Hillsboro, OR, USA) to confirm binding of the antibody-coated nanobeads to the *E. coli* O157:H7 cells. A sample was prepared using the protocol described in Section 2.5. Fixation was done using Karnovsky’s fixative followed by dehydration with successive ethanol washes.

### 2.10. Comsol Simulation

Comsol Multiphysics software was used to construct a computer model of the biosensor system. Simulations were performed to observe the effect of the *E. coli* cells on impedance change at varying distances from the electrode surface. A simplified computer model representing a 200 μm × 600 μm cutout of the electrode contained two 200 μm square electrode fingers separated by a 200 μm gap. A height of 150 μm was added to the system to simulate part of a droplet on the electrode surface. The layout of the computer model is shown in the supplemental material (Figure S2).

*E. coli* cells in the system were modeled as ellipsoids 1.5 μm long and 0.5 μm wide with membrane conductivity and cell wall permittivity of 0.5 S/m and 60, respectively [24,25]. For the interior of the chamber the conductivity and relative permittivity were set to 0.0001 S/m and 77 to simulate the 0.01 M PBS used in the experiment. The electrical conductivity of the gold electrode was set to 4.1 × 10^7^ S/m. The relative permittivity of gold is presumed to be infinite. Due to software limitations involved in solving for an infinite relative permittivity, boundary conditions were set for the surface of the gold electrode and the interior electrostatics were not solved for.

A voltage amplitude of 50 mV was applied at a frequency of 100 Hz and an electric field distribution was obtained, from which the impedance value was calculated. The simulated *E. coli* cells were positioned on the electrode surface as well as 1, 5, 10, 25, 50, and 75 μm above the electrode surface to observe the change in impedance values at different distances between the cells and electrode surface.

## 3. Results and Discussion

### 3.1. Characterization of Impedance Spectrum Data

**Figure 2:**
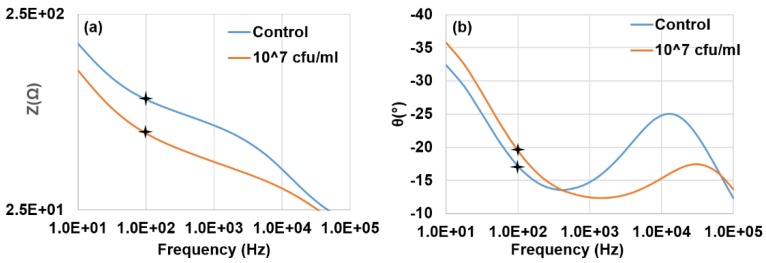
A typical Bode plot of the measured impedance data of the control and *E. coli* O157:H7 at a concentration of 10^7^ cfu/mL. (**a**) Impedance magnitude and (**b**) Phase angle. The frequency range was 10 Hz to 100 kHz. The amplitude of voltage applied was 50 mV.

Impedance magnitudes and phase angles for the detection of a sample containing 10^7^ cfu·mL^−1^ *E. coli* O157:H7 and a negative control sample are shown in Figure 2. The presence of bacteria resulted in a decrease in impedance magnitude and the maximum decrease occurred at 100 Hz. The phase angle describes the contribution of the resistance and capacitance elements to the impedance value. A current passing through a capacitor is phase shifted by −90° with respect to the voltage while a current passing through a resistor is in phase with the voltage, therefore having a phase angle of 0°. A phase angle between −90° and 0° indicates that the impedance value is affected by a combination of resistance and capacitive elements. The phase angles for both the bacterial and control samples decreased in the middle frequency around 500 Hz to 1 kHz and in the high frequency range nearing 100 kHz. In the higher frequency range between 10 kHz and 30 kHz the phase angle for both samples increased, though the bacterial sample phase angle was lower than the control sample’s. At the lower frequency range (10 Hz to 500 Hz), the phase angle of the bacterial sample was higher than the control sample’s. The phase angle data suggests that the presence of bacteria disrupted a capacitance element at the higher frequencies while creating a capacitance element in the low frequency range. An equivalent circuit model was built and evaluated to better understand the impedance spectrum data.

**Figure 3:**
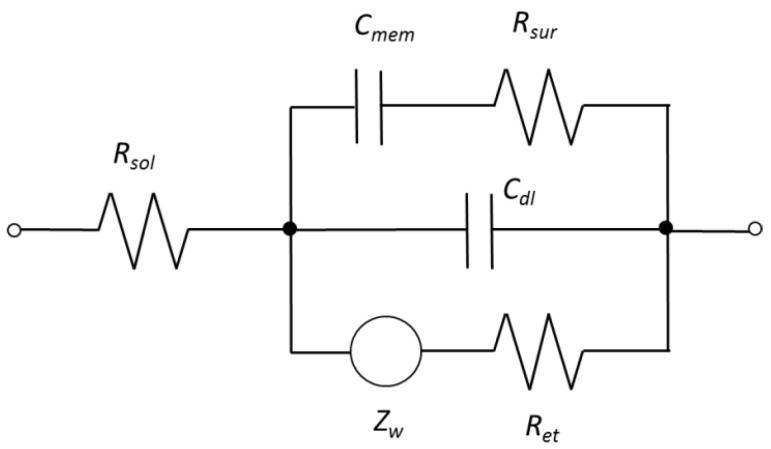
Equivalent circuit used for data analysis. The equivalent circuit components were bulk electrolyte (*R_sol_*), electron transfer resistance (*R_et_*), resistance due to surface roughness (*R_sur_*), double layer capacitance (*C_dl_*), capacitance of bacterial cells (*C_mem_*), and a Warburg impedance element (*Z_w_*).

The equivalent circuit shown in Figure 3 contained three resistance elements corresponding to bulk electrolyte resistance (*R_sol_*), electron transfer resistance (*R_et_*) and resistance due to surface roughness (*R_sur_*), two capacitance elements corresponding to double layer capacitance (*C_dl_*) and capacitance of bacterial cells (*C_mem_*)^25^, and a Warburg impedance element (*Z_w_*). A fitting analysis showed that the equivalent circuit fit the measured data with an average error of 0.4% and a maximum error of 4.6% for the impedance magnitude and an average error of 0.2° and a maximum error of 3.1° for the phase angle as shown in the supplemental materials (Figure S3).

Two capacitance elements, *C_mem_* and *C_dl_*, were looked at to better understand the phase angle data. The *C_dl_* element decreased by 53 nF between the control and the sample containing 10^7^ cfu·mL^−1^ cells, showing that the presence of bacteria disrupting the formation of a double layer capacitor on the electrode surface. The *C_mem_* element increased 13 μF between the control and the sample containing 10^7^ cfu·mL^−1^ cells, suggesting that the presence of the bacterial cells formed a capacitance element in the system [26]. From this information, it can be implied that the presence of bacteria disrupted the double layer capacitance in the high frequency range while producing a capacitance element in the low frequency ranges. This would explain the pattern seen in the phase angle data.

The *R_sur_* element decreased 76 Ω between the control and the sample containing 10^7^ cfu·mL^−1^ cells. The decrease in resistance may be explained by an increase in the electrode surface roughness due to the presence of nanobeads and nanobead/bacteria clusters on the electrode. The addition of the beads and clusters likely had the effect of increasing the conductive surface area of the electrode, thereby reducing the resistance of electrical flow. The nanobead/bacteria clusters may have also formed “bridges” between the electrode fingers, further reducing the resistance. The resistance of the electron transfer decreased 21 Ω between the control and the bacteria sample, indicating the bacteria did not form a large enough layer on the electrode surface to impede electron transfer from the surface of the electrode. *R_sol_* only decreased 3 Ω between the bacterial and control samples.

### 3.2. Detection of E. coli O157:H7

**Figure 4:**
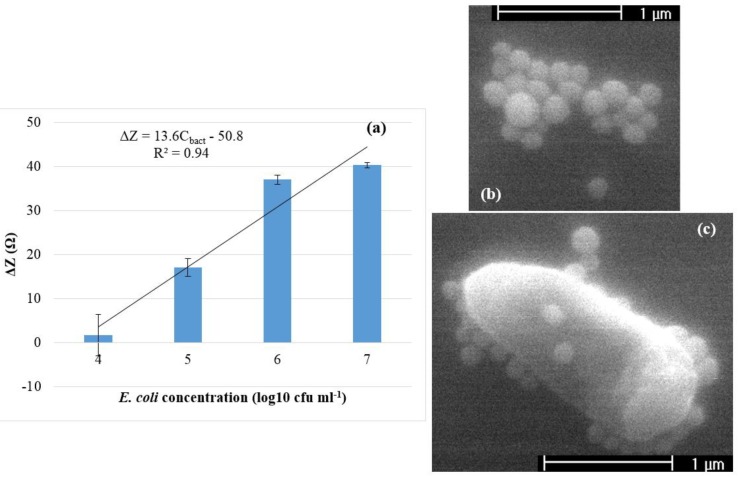
(**a**) Average impedance change between the control and bacteria measurements at 100 Hz for *E. coli* O157:H7. Error bars were based on standard deviation of triplicate tests; (**b**) ESEM photograph of the antibody-coated nanobeads; and (**c**) ESEM photograph of an *E. coli* O157:H7 cell attached with antibody-coated nanobeads.

The impedance magnitude at 100 Hz was determined to be the best indicator of bacterial presence. Figure 4a shows the average impedance decrease for each *E. coli* O157:H7 concentration. Based on the *t*-tests results, the *P*-value of impedance change (ΔZ) between 10^4^ and 10^5^ cfu·mL^−1^, 10^5^ and 10^6^ cfu·mL^−1^, and 10^6^ and 10^7^ cfu·mL^−1^ was 0.04, 0.002 and 0.05, respectively, indicating a significant difference. A linear relationship (*R*^2^ = 0.94) was found to exist between log value of *E. coli* concentration (*C*_bact_) in cfu·mL^−1^ and impedance change (ΔZ) in ohms between control and bacteria samples that corresponded to Δ*Z* = 13.6*C*_bact_ – 50.8. The lower detection limit was calculated to be 10^4.45^ cfu·mL^−1^. This corresponded to a final bacteria count of ~1400 cells in the applied volume of 25 µL. The reproducibility of the biosensor was shown to be high, with small standard deviations (SD = 2 ± 1) at all bacteria concentrations. The capture of the bacteria by the antibody-coated nanobeads was confirmed by ESEM, shown in the Figure 4c. To improve the specificity of the immunosensor, the combination of monoclonal antibodies against unique epitopes on the O157 and H7 antigens is a logical approach for specific detection of *E. coli* O157:H7, minimizing interferences from other bacteria including non-O157 and non-H7 serotypes of *E. coli*.

The *Z* and Δ*Z* values were in the low Ω range due to the use of 5 mM [Fe(CN)_6_]^3−/4−^ (1:1 ratio) mixture in PBS as a measurement solution. The use of this high electrolyte solution was needed to provide stability to the biosensor. Previous tests with low electrolyte solutions had very low repeatability. It was hypothesized that the diffusion of ionic species from the interior of the bacteria was affecting these tests and so a high electrolyte solution was used to negate the effect of those ions. This resulted in lower Z and ΔZ values but much higher repeatability.

The sensitivity of the biosensor could be potentially be improved by removing the free beads from the measurement sample. The presence of the free beads introduced noise into the system which may obscure the impedance measurement of *E. coli* O157:H7 at lower concentrations. Removal of free beads could be accomplished using a magnetophoretic separation device as described by Huang *et al*. [27]. Another way to improve the sensitivity is to apply further concentrated sample. In this study, a 2-times concentrated sample (from 200 µL sample to be concentrated to 100 µL) was used in all the tests. We conducted a quick test by applying 3-times concentrated sample, which resulted in a 16.7% increase in Δ*Z* value for *E. coli* O157:H7 at the concentration of 10^5^ cfu·mL^−1^, indicating that the magnetic nanobeads based isolation/concentration procedure could improve the sensitivity of the immunosensor. In order to detect *E.coli* at low concentrations, a more concentrated sample is needed.

While a reduction in the initial amount of nanobeads used in the test would likely reduce the noise of the system, previous unpublished research has shown that a reduction in the amount of nanobeads used in the test would result in a loss of capture efficiency. The data for these tests is included in the supplemental materials (Figure S4).

**Figure 5:**
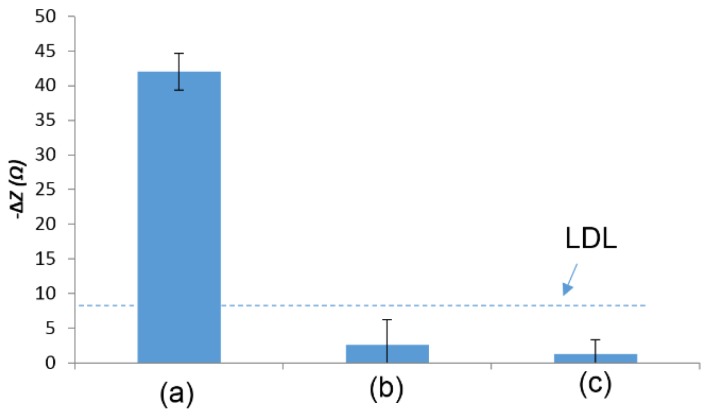
Average impedance change (**a**) between a control sample and nanobeads captured *E. coli* O157:H7 with a magnet underneath; (**b**) pure *E. coli* O157:H7; (**c**) between a control sample and nanobeads captured *E. coli* O157:H7 with no magnet underneath. *E. coli* O157:H7 concentration was 10^7^ cfu·mL^−1^. Error bars were based on standard deviation of triplicate tests. LDL line was determined by signal/noise ratio of 3, where noise was defined as the standard deviation of the pure redox probe measurements.

The magnetic nanobeads were determined to be necessary for detection by both experimental analysis and computer simulation. *Escherichia coli* cells without magnetic nanobeads were suspended in a redox probe solution and a drop of the sample was placed on a screen-printed electrode and the impedance was measured after 10 min. No detectable signal could be seen for *E. coli* cells only in a redox probe. Also *E. coli* samples were prepared as described in Section 2.5 but no magnetic field was applied after placing the samples on the electrode. Again, no detectable signal was seen. This data is included in Figure 5. Without a magnet underneath the electrode, the bacteria/nanobead complexes were loosely suspended in the drop solution without tight attachment onto the electrode surface, which might have negligible effect on the electrode surface. Based on the equivalent circuit shown in Figure 3 used for data analysis, the *R_sur_* element (electrode surface roughness) contributed the majority of the impedance change. When the magnet was applied underneath the electrode, the bacteria/nanobead complexes were tightly attached onto the electrode surface, which could increase the electrode surface roughness, resulting in a change in impedance. A simulation using Comsol Multiphysics (Figure 7) also showed that the impedance change decreases as the distance between the *E. coli* cells and electrode increases.

**Figure 6:**
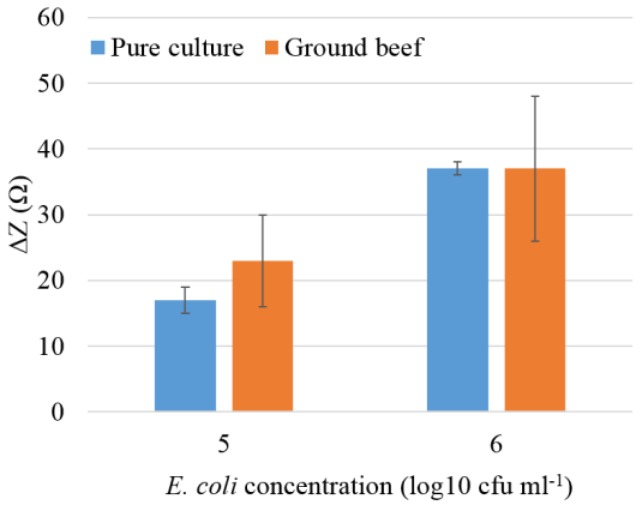
The comparison of impedance signals between the pure culture of *E. coli* O157:H7 and the ground beef with *E. coli* O157:H7 at concentrations of 10^5^ and 10^6^ cfu·mL^−1^ using the immunosensor.

To demonstrate the application of the immunosensor for food safety, food sample test was conducted using ground beef, which was inoculated with target bacteria of *E. coli* O157:H7 at concentrations of 10^5^ and 10^6^ cfu·mL^−1^. As shown in Figure 6, the change in impedance in ground beef sample that was artificially contaminated with *E. coli* O157:H7, was compared to the pure culture at the same concentration. The inoculated beef sample showed a detectable impedance response at both concentrations of 10^5^ and 10^6^ cfu·mL^−1^, which was comparable to the pure culture detection signal. However, larger error bars were observed in detection of ground beef than the pure culture. It was possibly due to the interference of the background of food samples. There is an abundant amount of protein, fat, and other components of meat products presented in ground beef.

### 3.3. Simulation of Impedance Change

**Figure 7:**
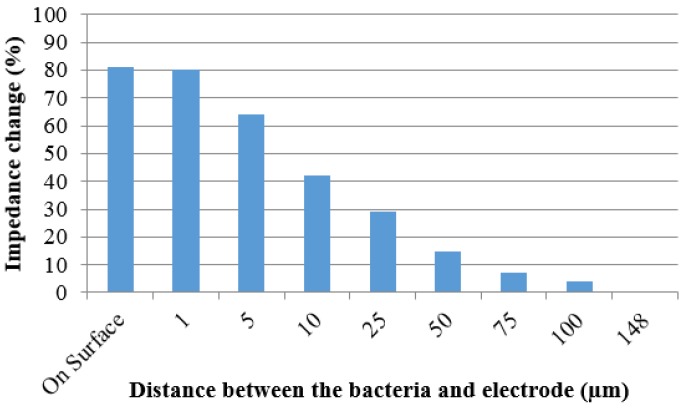
Impedance percent change due to simulated *E. coli* cells at varying distances from the electrode surface.

The simulation using Comsol Multiphysics showed that the impedance change decreases as the distance between the *E. coli* cells and electrode increases, as shown in Figure 7. When the *E. coli* cells are within 25 µm of the electrode surface there is a significant impedance change. When the *E. coli* cells are positioned on the electrode or just 1 µm above it the impedance change is 80%. Moving the *E. coli* cells out to 5, 10, or 25 µm away from the electrode resulted in the impedance change being decreased to 64%, 42%, and 29%, respectively. At distances greater than 25 µm the impedance change steadily decreases until 150 µm, where the same characteristics as if no cells are present. This may be explained by the cells being outside of the capacitive area, reducing their effect on the impedance value.

## 4. Conclusions

In this study an impedance immunosensor was developed for the rapid detection of *E. coli* O157:H7 using antibody-coated magnetic nanobeads and screen printed interdigitated electrodes. The antibody-coated magnetic nanobeads were shown to serve multiple roles in this impedance biosensor, including the isolation and concentration of the target bacteria from a sample and the placement of bacteria onto the electrode surface. The impedance biosensor was capable of detecting ~1400 cells of *E. coli* O157:H7 in less than 1 h.

## Supplementary Files

Supplementary File 1
