# Supplementary material for: A Label-Free Impedance Immunosensor Using Screen-Printed Interdigitated Electrodes and Magnetic Nanobeads for the Detection of E. coli O157:H7
Source: Biosensors (Basel). 2015 Dec 15;5(4):791–803. doi: 10.3390/bios5040791 (PMC4697145; doi:10.3390/bios5040791)
Supplement: Supplementary File 1 [file biosensors-05-00791-s001.pdf]

OPEN ACCESS

biosensors

ISSN 2079-6374

www.mdpi.com/journal/biosensors/

Supplementary Information

A Label-Free Impedance Immunosensor Using Screen-Printed Interdigitated Electrodes and Magnetic Nanobeads for the Detection of *E. coli* O157:H7. *Biosensors* 2015, *5*, 791-803

Ronghui Wang [1], Jacob Lum [2], Zach Callaway [1], Jianhan Lin [3], Walter Bottje [4] and Yanbin Li [1,2,4,5,*]

[1] Department of Biological and Agricultural Engineering, University of Arkansas, Fayetteville, AR 72701, USA; E-Mails: rwang@uark.edu (R.W.); zcallawa@uark.edu (Z.C.)

[2] Cell and Molecular Biology Program, University of Arkansas, Fayetteville, AR 72701, USA; E-Mail: jlum@pacificvetgroup.com

[3] College of Information and Electrical Engineering, China Agricultural University, Beijing 100083, China; E-Mail: jianhan@cau.edu.cn

[4] Center of Excellence for Poultry Science, University of Arkansas, Fayetteville, AR 72701, USA; E-Mail: wbottje@uark.edu

[5] College of Biosystems Engineering and Food Science, Zhejiang University, Hangzhou 310068, China

* Author to whom correspondence should be addressed; E-Mail: yanbinli@uark.edu; Tel.: +1-479-575-2881; Fax: +1-479-575-2846.

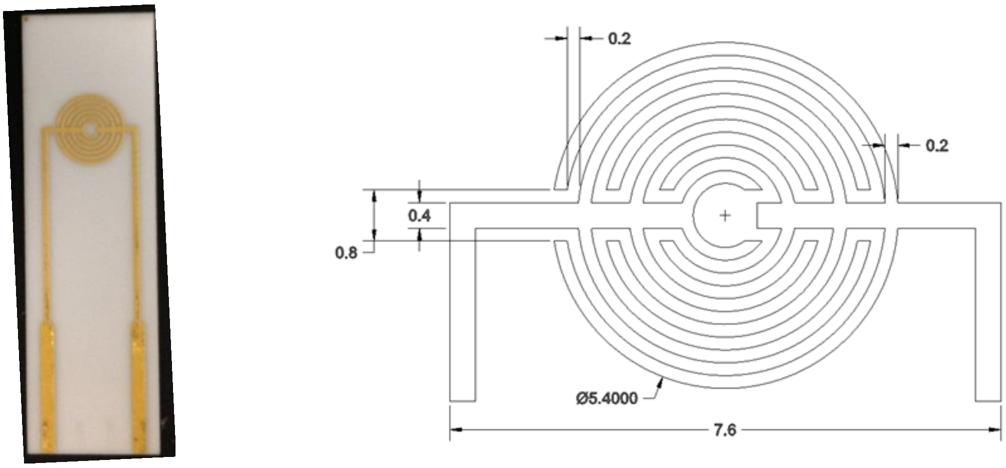

(**a**) (**b**)

Figure S1. (**a**) A photograph of the screen printed electrode and (**b**) drawing of the interdigitated electrode. Dimensions are given in millimeters.

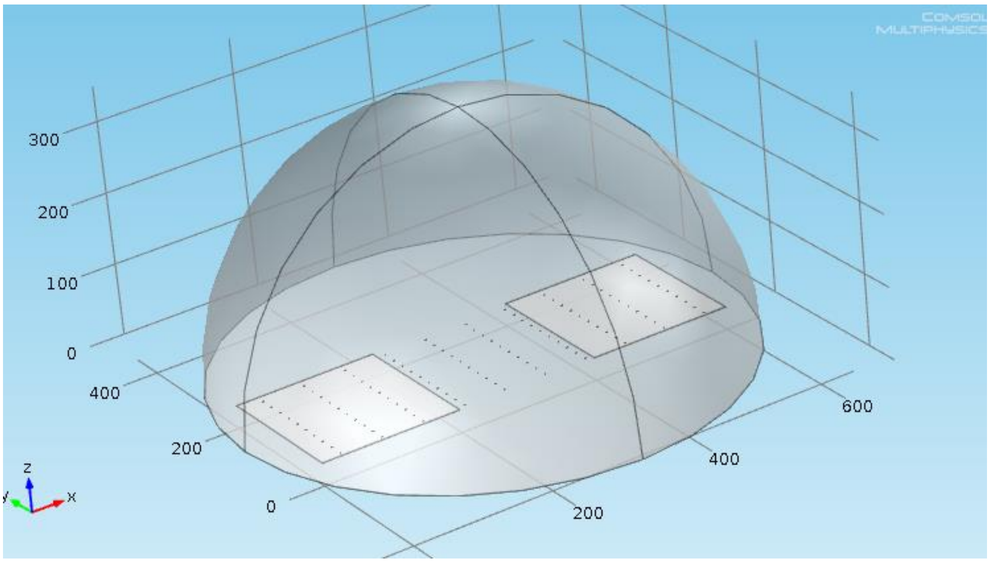

Figure S2. COMSOL model of simplified biosensor system with a pair of electrodes and 100 cells of *E. coli* evenly distributed on the electrode surfaces and the space between two fingers.

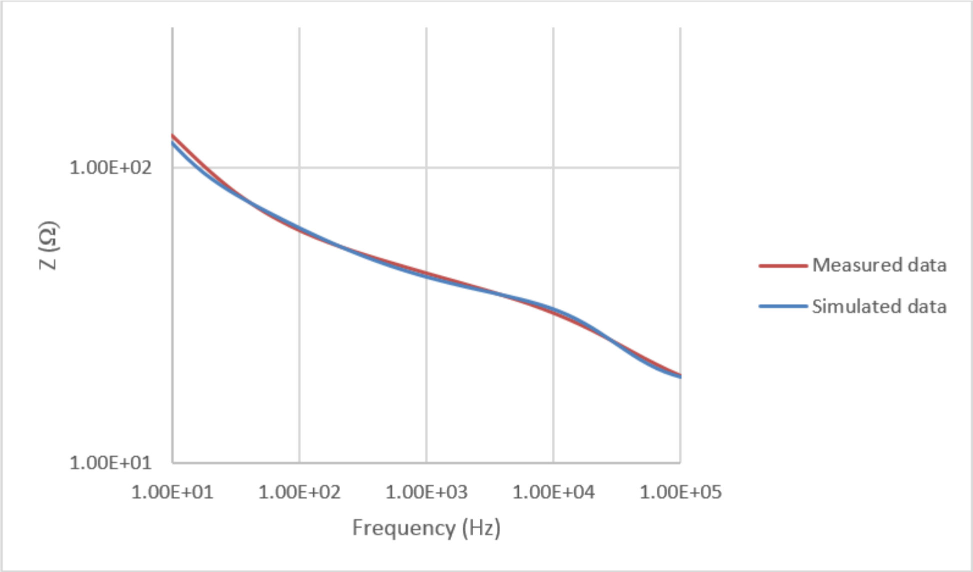

Figure S3. Bode diagram of measured impedance data and simulated impedance data generated by curve fitting of equivalent circuit. Measured data was taken with a sample containing 10^7 cfu mL^{-1} *E. coli* O157:H7.

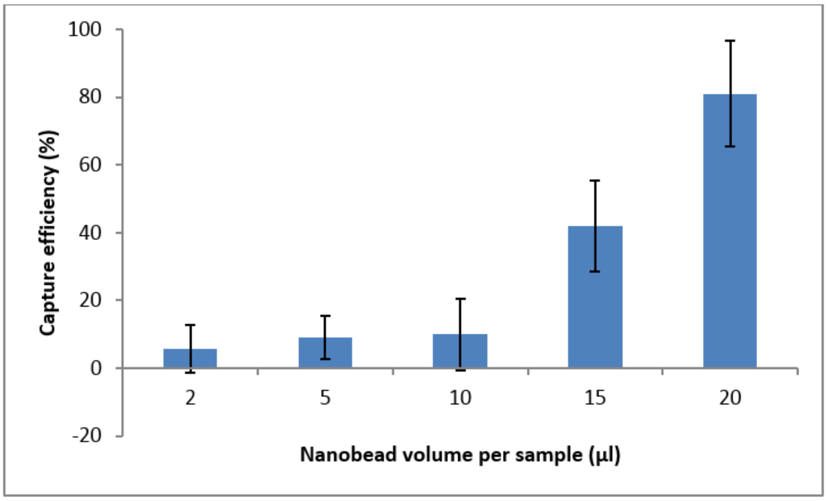

Figure S4. Capture efficiency of 10^5 cfu mL^{-1} *E. coli* O157:H7 using different amounts of antibody-coated magnetic nanobeads. The concentration of nanobeads was 1 mg mL^{-1}.

© 2015 by the authors; licensee MDPI, Basel, Switzerland. This article is an open access article distributed under the terms and conditions of the Creative Commons Attribution license (http://creativecommons.org/licenses/by/4.0/).
